# Methylprednisolone sodium succinate combined with azithromycin in the treatment of Mycoplasma pneumonia in children and Its effect on immune function

**DOI:** 10.3389/fped.2026.1842718

**Published:** 2026-09-22

**Authors:** Qingmei Zhang, Tingting Hao, Yifan Li

**Affiliations:** NO.215 Hospital of Shaanxi Nuclear Industry, Xi'an, China

**Keywords:** children, erythromycin, methylprednisolone sodium succinate, mycoplasma pneumoniae pneumonia, pneumoniae pneumonia

## Abstract

**Objective:**

To investigate the clinical efficacy of methylprednisolone sodium succinate combined with azithromycin in the treatment of pediatric bronchopneumonia, and to explore its comprehensive effects on immune regulation and inflammation relief.

**Methods:**

This retrospective controlled study included 120 pediatric patients with bronchopneumonia diagnosed in our hospital from October 2021 to March 2025. According to the treatment regimen, patients were divided into two groups: the experimental group (*n* = 60) received methylprednisolone sodium succinate combined with azithromycin on the basis of routine treatment, while the control group (*n* = 60) received azithromycin alone on the basis of routine treatment. The efficacy indicators before and after treatment, symptom improvement time (including time to defervescence, disappearance time of cough and sputum, resolution time of pulmonary rales, relief time of wheezing and dyspnea, time to disappearance of abnormal chest imaging, and length of hospital stay), inflammatory markers [interleukin-6 (IL-6), tumor necrosis factor-α (TNF-α)] and immune parameters [immunoglobulin A (IgA), IgG, IgM] of the two groups were analyzed, and the adverse drug reactions during treatment were recorded.

**Results:**

Compared with the control group, the overall efficacy of the experimental group was significantly better (total effective rate: 93.3% vs. 80.0%, *P* < 0.05). In terms of symptom improvement time, including time to fever resolution, disappearance of cough and sputum, resolution of pulmonary rales, relief of wheezing and dyspnea, disappearance of abnormal chest imaging and length of hospital stay, all indicators were significantly shortened in the experimental group (*P* < 0.05). After treatment, IL-6 and TNF-α levels were significantly decreased and IgA, IgG, and IgM levels were significantly increased in both groups, with more significant improvements observed in the experimental group (*P* < 0.05). The incidence of adverse drug reactions was similar between the two groups (8.3% vs. 13.3%, *P* > 0.05).

**Conclusion:**

Methylprednisolone sodium succinate combined with azithromycin can effectively shorten the course of mycoplasma bronchopneumonia in children, improve clinical symptoms, reduce inflammatory responses, and enhance immune responses. Under the premise of ensuring safety, it has advantages over azithromycin alone and has good clinical application prospects.

## Introduction

Pneumonia caused by Mycoplasma pneumoniae is classified as a respiratory disease. In recent years, the incidence of this disease in community-acquired pneumonia among children has continued to rise, becoming one of the leading causative factors of lobar pneumonia in children (1). The clinical manifestations of mycoplasma infection are diverse and often chronic in nature. Some cases may progress to severe pneumonia or induce immune-related complications such as bronchiolitis obliterans and interstitial lung disease, posing a significant threat to the health of affected children (2). Typical manifestations of pediatric Mycoplasma lobar pneumonia include persistent fever, severe cough, and diffuse shadows visible on chest x-ray. The condition progresses rapidly, and antibiotic monotherapy shows variable efficacy (3). As a representative macrolide antibiotic, azithromycin blocks the proliferation of Mycoplasma by inhibiting protein synthesis (4), making it the first-line drug for clinical treatment. However, with the widespread spread of resistant strains and the increasingly recognized role of immune-mediated mechanisms in the pathogenesis, antibiotic monotherapy has become less effective in controlling moderate to severe pediatric cases (5).

Previous studies (6) have indicated that Mycoplasma infection not only directly damages the upper respiratory tract and lung tissue, but also triggers abnormal immune responses, leading to elevated inflammatory cytokines, thereby exacerbating pulmonary inflammation and impairing lung function. Therefore, in the treatment of moderate to severe Mycoplasma pneumonia, the combined use of glucocorticoids with immunosuppressive and anti-inflammatory properties has become a new strategy. Methylprednisolone sodium succinate is a short-acting glucocorticoid with rapid and broad-spectrum anti-inflammatory and immunomodulatory effects. It can effectively inhibit cytokine release and regulate immune cell activity (7), thereby quickly controlling inflammation, alleviating pulmonary consolidation, and improving oxygenation. In recent years, some researchers have advocated the use of combined treatment strategies during the acute phase, combining glucocorticoids with other therapeutic agents to achieve faster and more pronounced therapeutic effects, while possibly reducing the dosage and duration of glucocorticoids, thereby minimizing long-term side effects (8, 9). Based on this concept, this study aimed to retrospectively evaluate the clinical efficacy of methylprednisolone sodium succinate combined with azithromycin in the treatment of pediatric mycoplasma alveolar pneumonia, focusing on its effects on symptom relief, immune function modulation, and inflammation suppression, so as to provide evidence for optimizing clinical treatment strategies for Mycoplasma pneumonia in the pediatric population.

## Materials and methods

### Study subjects and design

This study was a retrospective controlled trial aimed at evaluating the efficacy of methylprednisolone sodium succinate combined with azithromycin in the treatment of pediatric bronchopneumonia. The study strictly followed the ethical principles of the Declaration of Helsinki and was approved by the Ethics Committee of the hospital (Ethics Approval No.: 2021Y039). Informed consent was signed by all guardians of the children. Inclusion criteria were: (1) bronchopneumonia confirmed by serological testing (MP-IgM positive) and chest imaging; (2) age between 1 and 12 years; (3) disease duration ≤ 7 days with clinical manifestations consistent with bronchopneumonia; (4) conscious at admission and with moderate to severe pulmonary inflammation; (5) guardians signed informed consent and agreed to cooperate with the study protocol. Exclusion criteria: (1) concurrent other types of infections (bacterial, viral, or mixed infection); (2) history of chronic lung disease or immune system disorders; (3) recent use of glucocorticoids or immunosuppressants; (4) severe dysfunction of heart, liver, or kidney; (5) discontinuation during hospitalization or incomplete clinical data. A total of 120 pediatric patients diagnosed with bronchopneumonia in the Department of Respiratory Medicine of our hospital from October 2021 to March 2025 were included. According to the treatment received after admission, the patients were divided into two groups: Control group (*n* = 60): in addition to routine treatment, azithromycin antibiotic therapy was administered; Experimental group (*n* = 60): in addition to routine treatment, methylprednisolone sodium succinate combined with azithromycin was administered. There were no statistically significant differences in gender, age, disease course, lesion site, or disease severity between the two groups (*P* > 0.05), indicating comparability, as shown in Table 1.

**Table 1:** Comparison of baseline characteristics [$\bar{x}\pm s$, *n* (%)].

Characteristic	Control Group (*n* = 60)	Experimental Group (*n* = 60)	*t/x^2^*	*P*
Gender	-	-	0.135	0.712
Male	33 (55.0)	35 (58.3)	-	-
Female	27 (45.0)	25 (41.7)	-	-
Age (years)	7.35 ± 2.39	7.02 ± 2.51	0.737	0.462
Disease duration (days)	4.14 ± 1.17	4.05 ± 1.28	0.402	0.688
Lesion site	-	-	0.391	0.531
Left lung	22 (36.7)	21 (35.0)	-	-
Right lung	24 (40.0)	22 (36.7)	-	-
Bilateral	14 (23.3)	17 (28.3)	-	-
Disease severity	-	-	0.363	0.546
Moderate	44 (73.3)	41 (68.3)	-	-
Severe	16 (26.7)	19 (31.7)	-	-

### Treatment methods

After admission, all patients received comprehensive symptomatic treatment, including oxygen inhalation, fluid infusion, expectorant, antipyretic, and nutritional support. Control group: in addition to routine care, patients received intravenous azithromycin lactobionate (Hunan Kelun Pharmaceutical Co., Ltd., CFDA approval No.: H43020027) twice daily at a dose of 20–30 mg/kg for 5 consecutive days, adjusted or switched to oral administration according to clinical condition. Experimental group: in addition to the above treatment, patients received intravenous methylprednisolone sodium succinate (China National Pharmaceutical Shengsheng Pharmaceutical Co., Ltd., CFDA approval No.: H20010098) once daily at a dose of 2 mg/kg for 5 consecutive days. Blood pressure, blood glucose, electrolytes, and ECG changes were closely monitored during treatment.

### Observation indicators

(1) Clinical efficacy evaluation: Post-treatment evaluation criteria were as follows: Significantly effective: clinical symptoms completely disappeared, body temperature returned to normal, and chest x-ray showed complete absorption of pulmonary infiltrates; Effective: symptoms significantly improved, body temperature normal, and imaging showed pulmonary shadow absorption rate > 60%; Ineffective: no improvement in symptoms and absorption rate of infiltrates < 40%. Total effective rate was calculated as: (number of significantly effective cases + number of effective cases)/total number of cases×100%.
(2) Symptom relief time: including time to fever relief, time to relieve cough and sputum, disappearance time of pulmonary rales, time to relieve wheezing and dyspnea, time to absorption of x-ray shadows, length of hospital stay, etc., all recorded uniformly by medical staff.
(3) Inflammatory marker detection: 3 mL of fasting venous blood was collected before and after treatment, centrifuged (3000 r/min g, 5 min), serum was detected by an automatic biochemical analyzer (Shenzhen Mindray Bio-Medical Electronics Co., Ltd., Model: BS-600), and enzyme-linked immunosorbent assay (ELISA) was used to determine serum IL-6 and TNF-α levels.
(4) Immune function indicators: 2 mL of fasting venous blood was collected before and after treatment, and serum IgA, IgG, and IgM levels were measured by immunoturbidimetry using a specific protein analyzer (Beckman Coulter Trading Co., Ltd., Model: IMMAGE 800).
(5) Adverse reaction monitoring: Adverse events such as nausea, vomiting, skin reactions, gastrointestinal discomfort, elevated blood glucose, and sleep disturbances were observed and recorded during treatment, and the incidence of drug-related adverse reactions in both groups was calculated.

### Statistical methods

All data were analyzed using SPSS 26.0 statistical software. Normally distributed data were expressed as mean ± standard deviation ($\bar{x}\pm s$). Differences between groups were analyzed using independent sample t-test, and paired t-test was used for intra-group comparison before and after treatment. Categorical variables were expressed as *n* (%) and compared using the chi-square test. All statistical tests were two-tailed, and *P* < 0.05 was considered statistically significant.

## Results

### Comparison of treatment effectiveness

In the control group (*n* = 60), there were 17 significantly effective cases, 31 effective cases, and 12 ineffective cases; in the experimental group (*n* = 60), there were 21 significantly effective cases, 35 effective cases, and 4 ineffective cases. The overall therapeutic effect was significantly better in the experimental group (total effective rate: 93.3% vs. 80.0%, *P* < 0.05), as shown in Figure 1.

**Figure 1:**
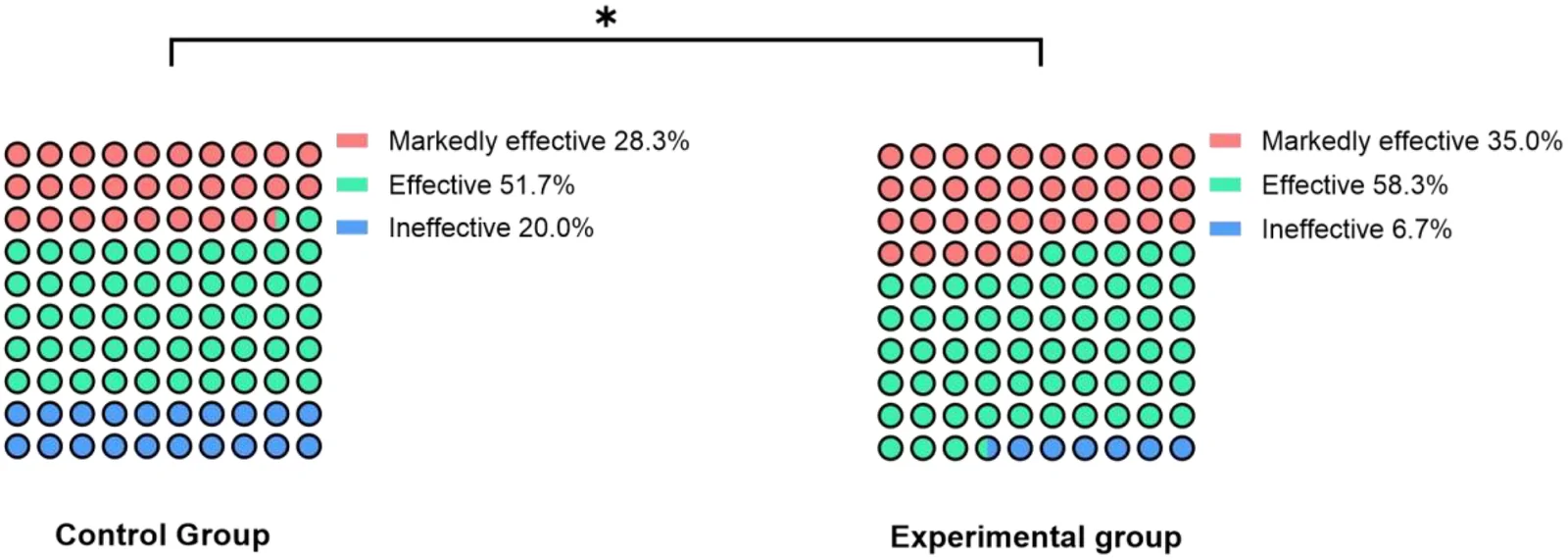
Comparison of treatment effectiveness [n (%)]. * indicates *P* < 0.05 between groups.

### Comparison of symptom relief time

Compared with the control group, the experimental group had significantly shorter times for fever reduction, disappearance of cough and sputum, pulmonary rales, wheezing, radiographic abnormalities, and length of hospital stay (*P* < 0.05), as shown in Figure 2.

**Figure 2:**
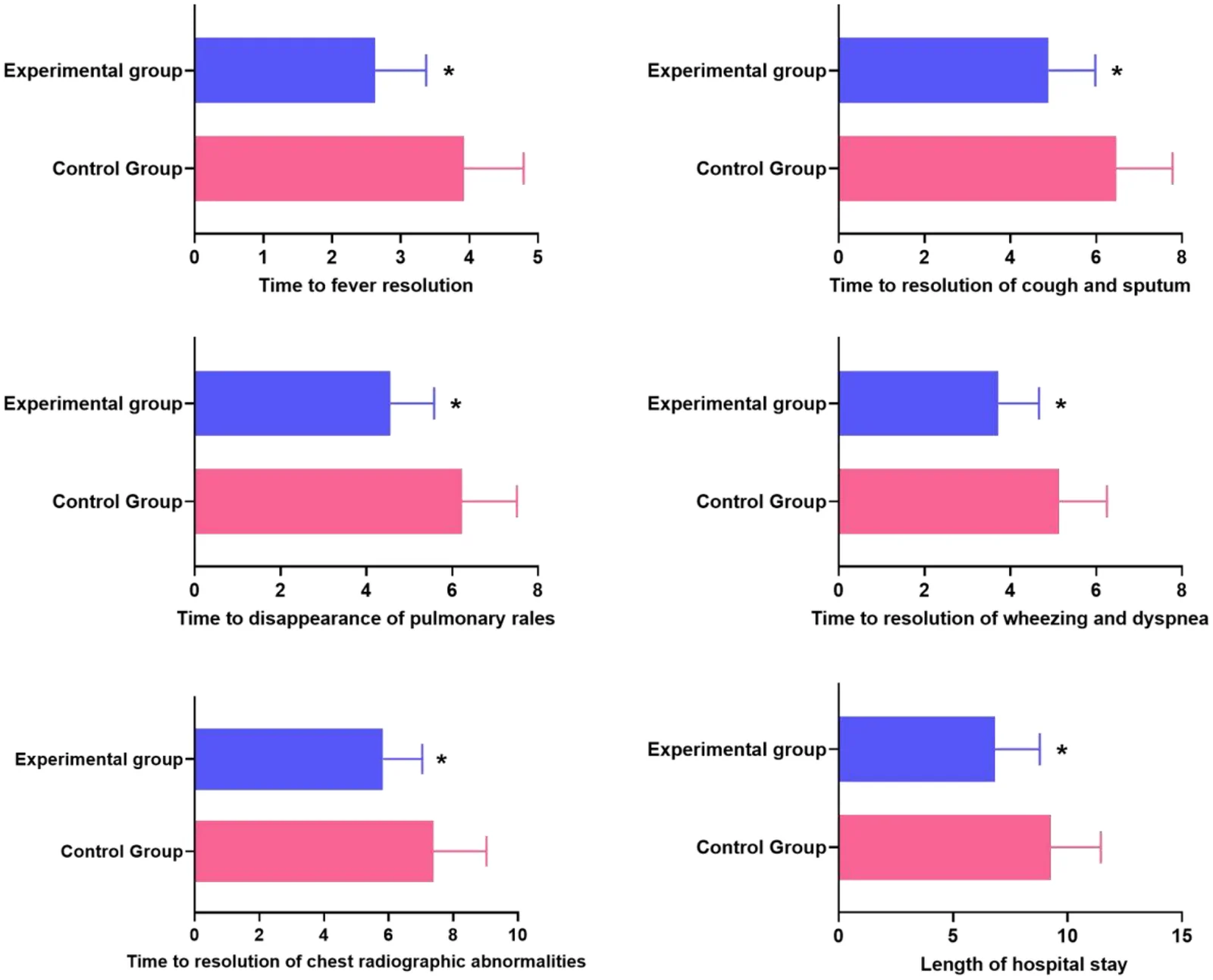
Comparison of symptom relief time (days). * indicates *P* < 0.05 between groups.

### Comparison of inflammatory markers

After treatment, IL-6 and TNF-α levels were significantly reduced in both groups compared with pre-treatment levels, with a greater improvement observed in the experimental group (*P* < 0.05), as shown in Table 2.

**Table 2:** Comparison of inflammatory markers ($\bar{x}\pm s$, pg/mL).

Marker	Control Group (*n* = 60)	Experimental Group (*n* = 60)	*t*	*P*
IL-6	-	-	-	-
Before treatment	55.92 ± 9.87	55.27 ± 10.01	0.358	0.720
After treatment	33.26 ± 6.72^a^	25.95 ± 5.48^a^	6.530	＜0.001
TNF-α	-	-	-	-
Before treatment	48.09 ± 9.12	49.33 ± 8.87	0.755	0.451
After treatment	31.84 ± 7.06^a^	22.97 ± 6.15^a^	7.338	＜0.001

a indicates *P* < 0.05 compared with Before treatment within the same group.

### Comparison of immune function indicators

After treatment, IgA, IgG, and IgM levels were significantly increased in both groups, with more pronounced improvements in the experimental group (*P* < 0.05), as shown in Figure 3.

**Figure 3:**
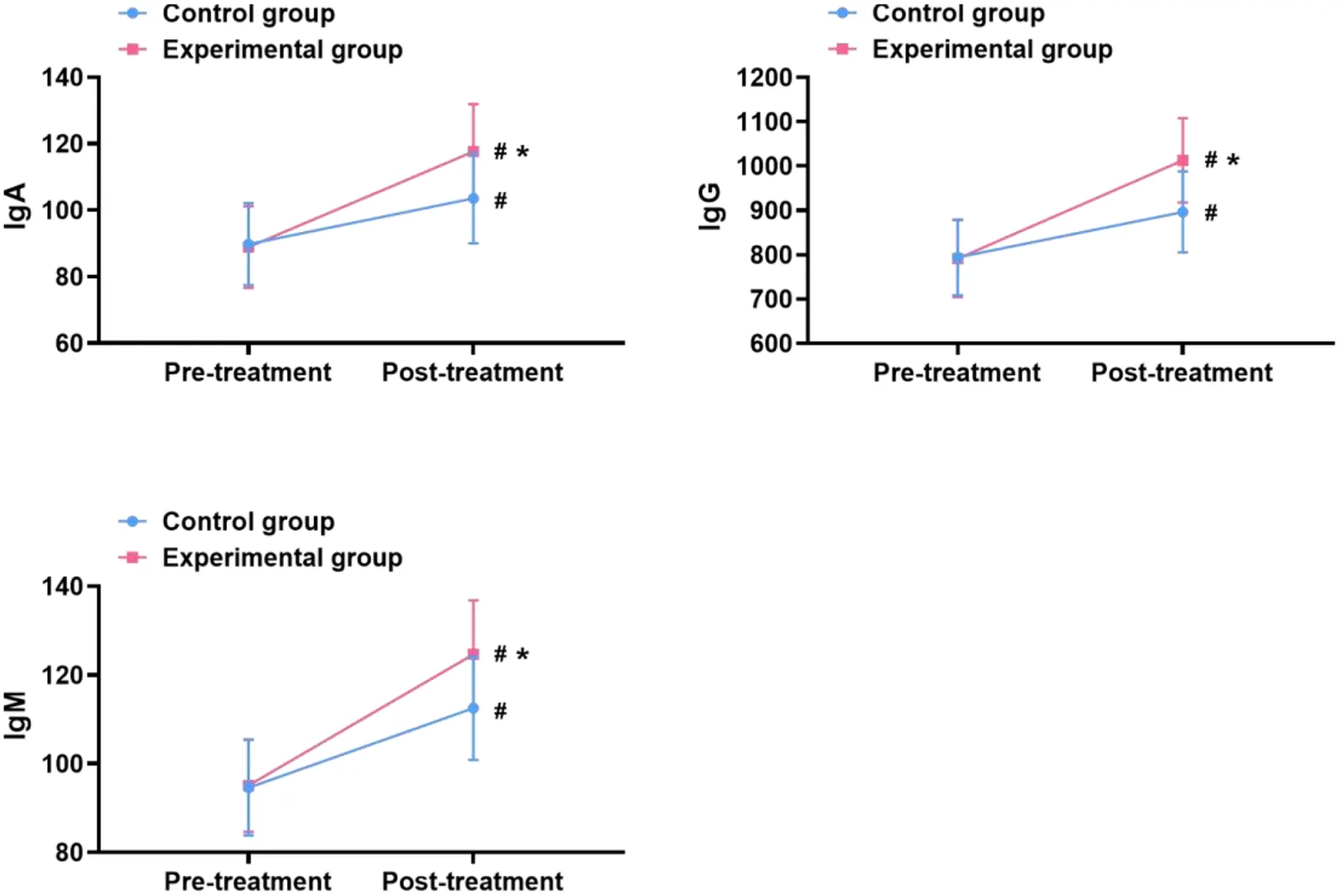
Comparison of immune function indicators (mg/dL). # indicates *P* < 0.05 compared with Before treatment within the same group; * indicates *P* < 0.05 between groups.

### Comparison of adverse reactions

As shown in Table 3, the incidence of drug-related adverse reactions was similar between the control group and the experimental group (8.3% vs. 13.3%, *P* > 0.05).

**Table 3:** Comparison of adverse reactions [n (%)].

Adverse reaction	Control Group (*n* = 60)	Experimental Group (*n* = 60)	*x^2^*	*P*
Nausea/vomiting	1 (1.7)	2 (3.3)	-	-
Skin reactions	1 (1.7)	1 (1.7)	-	-
Gastrointestinal discomfort	2 (3.3)	2 (3.3)	-	-
Hyperglycemia	0 (0.0)	2 (3.3)	-	-
Sleep disturbances	1 (1.7)	1 (1.7)	-	-
Total adverse incidence	5 (8.3)	8 (13.3)	0.830	0.362

## Discussion

Mycoplasma pneumoniae infection (especially lobar type) has a high incidence in the pediatric population and presents diverse clinical manifestations (10). With the increasing prevalence of drug-resistant mycoplasma strains and deeper insight into immune mechanisms, monotherapy with antibiotics alone shows limitations (11), prompting clinicians to explore multi-target intervention strategies to improve prognosis. This study evaluated the clinical value of methylprednisolone sodium succinate combined with azithromycin. The results confirmed that this regimen provides significant advantages in enhancing efficacy, relieving symptoms, controlling inflammatory responses, and improving immune function, demonstrating good clinical application prospects. Mycoplasma pneumoniae is a microorganism lacking a cell wall, and its pathogenesis depends not only on bacterial load but mainly on activation and dysregulation of the host immune response (12). Literature (13, 14) indicates that mycoplasma infection can activate dendritic cells and T lymphocytes, triggering Th1/Th17-mediated inflammatory responses and releasing large numbers of pro-inflammatory cytokines, leading to a cytokine storm that damages alveolar structures. In addition, in some patients, immune-mediated mechanisms remain activated even after pathogen clearance, resulting in persistent pulmonary inflammation. Such inflammatory responses are key drivers of sustained symptoms and disease worsening (15). Conventional treatment mainly relies on macrolide antibiotics such as azithromycin, which exert their effects by inhibiting bacterial protein synthesis. However, due to resistant mutations of mycoplasma or excessive immune reactions, the efficacy of azithromycin may be limited in some pediatric patients, and in certain cases may exacerbate the inflammatory response, worsening the condition (16). Therefore, incorporating anti-inflammatory glucocorticoids into the current treatment regimen has become an important strategy.

Methylprednisolone sodium succinate is a water-soluble glucocorticoid with excellent tissue penetration and significant immunomodulatory effects (17). This drug exerts rapid anti-inflammatory effects through both genomic and non-genomic pathways, with primary mechanisms including: (1) inhibiting synthesis of inflammatory mediators (such as prostaglandins and leukotrienes) and blocking inflammation-related signaling pathways (e.g., NF-*κ*B, MAPK) (18, 19); (2) reducing vascular permeability and limiting inflammatory cell infiltration; and (3) inhibiting activation of immune cells (T cells, macrophages) and release of cytokines (20). During the acute phase of mycoplasma pneumonia, short-term high-dose “combination therapy” can effectively control immune storms while avoiding long-term medication side effects (21). The results of this study showed that the reductions in inflammatory markers (IL-6, TNF-α) in the experimental group were significantly greater than those in the azithromycin-only group, confirming the advantage of combination therapy in inflammation control. In addition, azithromycin, as a broad-spectrum macrolide antibiotic, not only maintains strong inhibitory effect against mycoplasma but also exhibits certain immunomodulatory functions. Literature (22) indicates that azithromycin can alleviate airway inflammation by inhibiting neutrophil chemotaxis and reducing cytokine release. Therefore, its combination with methylprednisolone can synergistically regulate immune responses on the basis of antimicrobial effects, providing dual protection for inflammation control and lesion resolution.

In the pathogenesis of pediatric lobar mycoplasma pneumonia, pathogen stimulation and persistent inflammatory response place a heavy burden on the immune system, leading to varying degrees of immune dysfunction (23). Immunoglobulins are key indicators of humoral immunity: IgA is mainly responsible for mucosal defense, IgM is the responsive antibody in early acute infection, and IgG plays a central role in maintaining immune response and memory (24). This study found that some patients exhibited increased IgA and IgM but decreased IgG during the acute phase, suggesting impaired humoral immune regulatory function. Post-treatment observations demonstrated significantly increased IgA, IgG, and IgM levels in the experimental group, with improvements significantly superior to the control group. Considering the dual mechanisms of azithromycin's antibacterial action and methylprednisolone's immunomodulatory effects, it can be inferred that the combination therapy not only combats infection but also suppresses excessive inflammatory responses, thereby reducing the immune system's energy and resource burden, alleviating immune cell “fatigue” (25), and promoting gradual restoration of immune balance. Furthermore, short-term combined use of glucocorticoids can avoid persistent immunosuppression without interfering with normal immunoglobulin synthesis, consistent with literature reports that “appropriate glucocorticoid use may promote immune tolerance and reconstruction” (26, 27). Therefore, the combination of methylprednisolone and azithromycin not only possesses effective antibacterial action but also supports immune function, providing a theoretical and practical basis for immunomodulatory intervention in children with complex disease progression.

Pulmonary function impairment is one of the core pathological changes of Mycoplasma bronchopneumonia infection, mainly manifested as increased airway resistance, poor small airway ventilation, and decreased lung tissue elasticity. These changes can seriously affect gas exchange efficiency (28), thereby influencing the recovery speed and quality of life in affected children. This improvement may be related to multiple factors: first, azithromycin can effectively clear Mycoplasma infection and reduce pathogen load; second, methylprednisolone can inhibit infiltration of inflammatory cells and release of inflammatory mediators in the airway, thereby reducing airway edema and mucus secretion and improving airway patency (29). More importantly, glucocorticoids can suppress airway hyperresponsiveness, stabilize bronchial smooth muscle, and promote mucosal repair, accelerating pulmonary function recovery. Furthermore, pulmonary inflammation control and structural repair are interrelated processes (30). Effective anti-inflammatory intervention can not only alleviate current ventilatory limitation but also reduce the risk of airway remodeling and chronic pulmonary dysfunction. Therefore, the combination therapy regimen has important value in protecting and restoring pulmonary function in children, especially in cases involving extensive pulmonary involvement and severe ventilatory disorder.

In terms of safety, although glucocorticoid use in pediatrics has long been controversial—mainly due to concerns about inducing hyperglycemia, electrolyte imbalance, gastrointestinal discomfort, or affecting growth and development (31)—this study did not observe a significant increase in adverse events, indicating that methylprednisolone is safe and controllable when used at appropriate doses and treatment durations. It is recommended that clinicians strictly follow indications and treatment windows, especially in cases of persistent high fever, extensive pulmonary infiltrates, markedly elevated inflammatory markers, or poor effect of single antibiotic therapy. In addition, enhanced monitoring during medication (such as dynamic assessment of blood glucose, electrolyte levels, and ECG) helps to identify and intervene in potential risks early.

Multiple clinical studies have confirmed the value of glucocorticoids in moderate-to-severe Mycoplasma pneumonia infection (32, 33). For example, the study by Qiu et al. (33) showed that early combined glucocorticoid therapy in children with severe Mycoplasma pneumonia can significantly shorten the course of disease and reduce the incidence of pulmonary complications. This result is consistent with the conclusions of this study and provides further evidence from immunological and pulmonary function dimensions. However, it is important to note that large-scale, multicenter, long-term follow-up studies are still lacking to fully evaluate the long-term impact of this strategy on immune function and lung development. Future research should focus on the following directions: (1) optimizing individualized glucocorticoid dosage and treatment duration; (2) long-term impact of combination therapy on lung structural recovery; (3) integrating multi-omics markers such as inflammation, immunity, and microbiome to reveal mechanistic heterogeneity; (4) establishing clinical prediction models to guide precise application of combination therapy.

## Conclusion

This study demonstrates that the treatment regimen of methylprednisolone sodium succinate combined with azithromycin has significant clinical benefits for children with lobar Mycoplasma pneumonia. This therapeutic strategy not only significantly shortened the duration of clinical symptoms and promoted resolution of pulmonary lesions, but also effectively controlled systemic inflammatory responses, improved immune and pulmonary function indicators, and did not significantly increase the risk of drug-related adverse events, showing favorable safety characteristics. Mechanistically, the combination of antibiotics with short-course glucocorticoids forms a dual strategy: it not only suppresses pathogens but also rapidly alleviates excessive immune-inflammatory responses, providing an optimal therapeutic window for children with moderate-to-severe Mycoplasma pneumonia. In summary, the combination therapy of methylprednisolone sodium succinate and azithromycin is worthy of clinical promotion, especially for children with significant inflammatory responses, rapidly progressing disease, or poor response to antibiotic monotherapy. Future multicenter prospective studies are still needed to verify its long-term efficacy and mechanisms, so as to guide more precise and individualized treatment decisions.

## Data Availability

The original contributions presented in the study are included in the article/Supplementary Material, further inquiries can be directed to the corresponding author.
